# A Conspectus of Euvolemic Hyponatremia, Its Various Etiologies, and Treatment Modalities: A Comprehensive Review of the Literature

**DOI:** 10.7759/cureus.43390

**Published:** 2023-08-12

**Authors:** Anit Ghosal, Hafiza Amna Qadeer, Sravan K Nekkanti, Priyanka Pradhan, Chiugo Okoye, Danish Waqar

**Affiliations:** 1 Internal Medicine, Kolkata Medical College and Hospital, Kolkata, IND; 2 Internal Medicine, Foundation University Medical College, Islamabad, PAK; 3 Internal Medicine, Davao Medical School Foundation, Davao, PHL; 4 Internal Medicine, Igbinedion University, Okada, NGA; 5 Internal Medicine/Nephrology, Loyola University Medical Center, Chicago, USA

**Keywords:** diagnostic and therapeutic approach, etiology, adrenal insufficiency, syndrome of inappropriate antidiuretic hormone secretion, hyponatremia

## Abstract

Hyponatremia is the most prevalent electrolyte imbalance encountered among hospitalized patients, athletes, the elderly, patients with chronic ailments, postoperative patients, and a few asymptomatic individuals. Clinical manifestations of hyponatremia can be diverse, with characteristic neurological symptoms. Depending on in-depth medical history, physical examination (including volume status assessment), laboratory investigation, and drug history, patients can be classified broadly as undergoing hypervolemic, euvolemic, or hypovolemic hyponatremia. However, patients with hypervolemic hyponatremia often present with distinctive signs such as edema or ascites, and the clinical presentation of hypovolemic and euvolemic hyponatremia poses significant challenges for clinicians. The convolution in clinical manifestations of patients is due to the varied etiologies of euvolemic hyponatremia, such as syndrome of inappropriate antidiuretic hormone secretion (SIADH), adrenocortical insufficiency, hypothyroidism, psychogenic polydipsia, different classes of drugs (chemotherapeutics, antipsychotics, antidepressants), endurance exercise events, and reset osmostat syndrome (ROS). The management of hyponatremia depends on the rate of hyponatremia onset, duration, severity of symptoms, levels of serum sodium, and underlying comorbidities. Over the last decade, the clinical understanding of hyponatremia has been scattered due to the introduction of innovative laboratory markers and new drugs. This article will be a conspectus of all the recent advancements in the field of diagnosis, investigations, management, and associations of hyponatremia, along with traditional clinical practices. Subsequently, a holistic overview has been laid out for the clinicians to better understand and identify knowledge deficiencies on this topic.

## Introduction and background

Hyponatremia occurs when the serum sodium level is <135 mEq/L; such levels of sodium (Na) are commonly seen during hospital stays [1]. It is a pathophysiologic condition indicating a water homeostasis disruption [2]. Excessive fluid intake, certain medications, hormonal imbalances, kidney problems, heart failure, liver disease, and some underlying medical conditions are among the potential causes of hyponatremia [3]. In some cases, hyponatremia can also result from a combination of factors, often with the simultaneous presence of volume depletion, the syndrome of inappropriate ADH secretion (SIADH), and adrenal insufficiency [4]. The clinical manifestation of hyponatremia varies depending on the severity and rate of the decrease in plasma sodium concentration and any neurological disorders or other electrolyte abnormalities. In this context, mild hyponatremia can present with nausea, vomiting, headache, muscle weakness, and tiredness, but in severe cases, symptoms consist of confusion, seizures, and coma [3]. The diagnostic workup for hyponatremia involves the measurement of sodium levels in the blood as well as urine sodium, potassium, creatinine values, the total amount of body water, and, in some cases, hormone levels that influence water uptake by the kidneys [5]. The treatment of hyponatremia depends on the cause and severity of the condition. In some cases, simply reducing fluid intake may be sufficient. Other treatment options focus on addressing the underlying cause, adjusting medications, and administering intravenous fluids or medications to restore sodium levels. Many patients recover entirely from hyponatremia with adequate treatment. However, elderly individuals and those hospitalized for a long time have worse outcomes [5]. This review article aims to dive into the pathophysiology, etiology, clinical manifestation, diagnostic methodology, and therapeutic strategies of hyponatremia. We intend to contribute knowledge and guidance on managing hyponatremia in daily clinical practice by highlighting these factors based on current evidence.

## Review

Euvolemic hyponatremia secondary to the syndrome of inappropriate antidiuretic hormone secretion (SIADH)

Hyponatremia has multiple etiologies, but the most common cause, which constitutes 60% of all types, is the syndrome of inappropriate antidiuretic hormone secretion (SIADH) [6]. The syndrome of inappropriate antidiuretic hormone secretion is a condition in which the antidiuretic hormone level is abnormally high irrespective of low serum osmolarity, causing sodium and water imbalance depicted by hypoosmolar hyponatremia and impairment in urinary water excretion except for kidney disease or other endocrinology influences resulting in antidiuretic hormone (ADH) release [7,8]. It was first described by Schwartz et al. in 1957 in two patients with bronchogenic carcinoma with low serum osmolality and concentrated urine (i.e., >100 mOsm/kg H2O) [9]. An increase in plasma arginine vasopressin (AVP) is the single most critical factor in confirming the clinical diagnosis; however, the trend of the syndrome of inappropriate antidiuresis (SIAD) has transformed over the years. Not all patients have increased levels of plasma AVP, and any measured value of AVP can be deranged in the state of low serum osmolality, therefore being deemed "inappropriate" [10]. Hence, "syndrome of inappropriate antidiuresis (SIAD)" is a preferred term over SIADH. The syndrome of inappropriate antidiuresis has been associated with a variety of conditions. According to recently published articles, the most common causes of SIAD include tumors, pulmonary disease, central nervous system (CNS) disorder, and medication, although their prevalence may vary with every study. A large multicenter hyponatremia registry study consisting of European and U.S. populations revealed that tumors were a significant cause (24%), followed by drugs (18%), pulmonary disease (11%), and central nervous system (CNS) disease (9%) [11]. Furthermore, malignancy and medication were the most typical causes of SIAD, based on a retrospective study comprising 555 patients [9].

Hyponatremia secondary to SIADH occurs when the serum sodium level is <135 mmol/L. It is associated with significant mortality and represents a negative prognostic indicator [12,13]. The syndrome of inappropriate antidiuretic hormone secretion is the most common etiology of euvolemic hyponatremia [14], and the prevalence of the latter is roughly 2.4%, whereas severe hyponatremia (defined as a serum sodium level of <125 mmol/L) affects around 0.13% of hospitalized patients [13]. In particular, the prevalence of euvolemic hyponatremia due to SIADH is higher in frail elderly patients with fragility fractures (EPFF) [15]. The most common cause of SIADH in children is hypothalamic-pituitary dysregulation of ADH secretion, whereas, in adults, SIADH is primarily linked to paraneoplastic disorders characterized by ectopic production of ADH or related to neoplastic localizations in the lungs and central nervous system [16,17]. Conditions including bronchial carcinoma, acute respiratory failure, psychosis, meningitis, hydrocephaly, encephalitis, and spinal cord lesions may also be characterized by euvolemic hyponatremia secondary to SIADH [18-20]. Hyponatremia resulting from SIADH can also be a frequent complication during pulmonary infections (pneumonia) and central nervous system (CNS) infections, whereby the critical mechanisms involved in SIADH development are attributed to hypoxia and intravascular volume depletion [21,22]. Given that SIADH is more frequently observed in patients with greater disease severity as well as inflammation, it is plausible that nonosmotic stimulation of ADH secretion may result from nonosmotic stimulation of ADH secretion triggered by excessive amounts of pro-inflammatory cytokines released, especially interleukin 6 (IL-6) [21].

Adrenal Insufficiency vs. SIADH

Studies have shown that 2.7%-3.8% of patients who present to the emergency room with euvolemic hyponatremia have an undiagnosed underlying adrenal insufficiency, and it can reach up to 15% among those admitted to specialized care units [23]. The pathophysiology of hyponatremia in adrenal insufficiency has been well-described in the literature; cortisol deficiency causes increased corticotropin-releasing hormone (CRH) release from the hypothalamus, and CRH is an ADH secretagogue. Cortisol inhibits ADH, and cortisol deficiency removes the feedback inhibition of CRH, leading to increased ADH secretion [24]. Moreover, cortisol alters the permeability of the collecting duct to water and is needed for free water excretion from the kidneys. Early studies showed that in rats with inherited diabetes insipidus (DI), adrenalectomy led to impaired renal water clearance, which was restored when prednisolone and aldosterone were administered [25,26]. Essentially, the presence of ADH despite hypoosmolality and impaired renal collecting tubule permeability to water plays a role in causing hyponatremia in adrenal insufficiency.

One of the causes of euvolemic hyponatremia is SIADH, as mentioned earlier, whereby the ADH secretion remains high despite the low osmolality, resulting in high urine osmolality and natriuresis; the serum osmolality is less than 275 mOsm/kg (normal range: 275-295 mOsm/kg); the urine electrolytes are significant for urine sodium >40 meq/L; and the urine osmolality is >100 mOsm/kg, usually reaching more than 300 mOsm/kg. Similar features are also seen in hyponatremia resulting from cortisol deficiency. Hence, it becomes essential to rule out cortisol deficiency before diagnosing SIADH in a patient. There are several causes of adrenal insufficiency (AI), including pathology of the adrenal gland (primary adrenal insufficiency (PAI)), hypothalamic or pituitary pathology (secondary adrenal insufficiency (SAI)), or suppression of the hypothalamic-pituitary-adrenal (HPA) axis by exogenous glucocorticoids [27]. Interestingly, SAI causes a SIADH-like picture mostly because ADH release in Addison's disease or primary adrenal insufficiency is "appropriate" because of salt and water wasting due to mineralocorticoid deficiency. The syndrome of inappropriate antidiuretic hormone secretion has unique features that can differentiate it from other causes of hyponatremia. Since both causes of hyponatremia (SIADH and adrenal insufficiency) have similar laboratory investigation outcomes, it can be challenging to rule out adrenal insufficiency as a cause of hyponatremia. Confirmation of the clinical diagnosis of adrenal insufficiency is mainly a three-stage process:

A) Demonstrating the presence or absence of inappropriately low cortisol secretion

B) Determining whether the cortisol deficiency is dependent on or independent of corticotropin (ACTH).

C) Seeking a treatable primary or secondary disorder cause [28].

Once the diagnosis is confirmed, treatment for adrenal insufficiency primarily depends on the etiology. Mainly, the treatment is based on fulfilling the cortisol deficiency by giving cortisol (with IV hydrocortisone or oral prednisone). Intravenous hydrocortisone or oral prednisone increases cortisol levels, suppressing the CRH and subsequently normalizing ADH secretion, leading to the resolution of hyponatremia.

Hyponatremia Associated with Hypothyroidism

Up to 10% of hypothyroid patients have hyponatremia, although it usually occurs mildly and seldom results in symptoms. The most significant mechanism of hyponatremia in acute hypothyroidism is a decreased glomerular filtration rate (GFR), which can directly reduce free water excretion by reducing water transport to the diluting segments of the kidney [29,30]. In patients with chronic hypothyroidism, the leading cause of hyponatremia is a diminished ability to excrete free water due to high ADH [31]. Levothyroxine therapy corrects ADH levels in cases of hypothyroidism, as it is associated with decreased GFR and renal plasma flow [32]. Some hypothyroid patients have higher sodium concentrations in their urine, which may lead to a misdiagnosis of SIADH [33]. Other comorbidities, such as hypovolemia, nausea, infections, or medications that impact water homeostasis, causing increased ADH secretion and water retention, may at least be partially responsible for the association between hyponatremia and hypothyroidism [34,35].

Psychogenic Polydipsia

Hyponatremia can also result from an underlying pathological condition such as psychogenic polydipsia, a habit of excessive water drinking, especially in a population with a background of psychiatric disorders. It can be associated solely with psychiatric conditions themselves or might be due to certain medications, leading to symptoms of confusion, lethargy, psychosis, seizures, or death [36]. Excess water consumption in patients with psychiatric disorders, especially those with schizophrenia, is potentially attributed to an impairment in the thirst center in the hypothalamus, predisposing them to hyponatremia, which is not preferred in these patients who are already acutely psychotic. It has come to light that a few antipsychotic medications could be the root cause, prompting these patients to develop psychogenic polydipsia [37]. Psychogenic polydipsia can be managed by restricting fluid intake and switching to alternative drugs that do not lead to anticholinergic effects [36].

Idiosyncratic Drug Reaction Resulting in Hyponatremia

Euvolemic hyponatremia secondary to SIADH occurs following the use of certain drugs that influence the sodium-water homeostasis of the body, such as anticonvulsants (carbamazepine), antineoplastic agents, antidepressants (tricyclic antidepressants, selective serotonin reuptake inhibitors), anti-Parkinson drugs, oral hypoglycemic agents, lipid-lowering agents, angiotensin-converting enzyme (ACE) inhibitors, loops diuretics, thiazide diuretics, or amiloride [18,19,20]. On the other hand, drugs that do not primarily manipulate the sodium-water homeostasis but lead to hyponatremia likely increase the ADH in the blood (irrespective of the hypotonicity of the serum), most frequently caused by SIADH or effective circulating volume depletion (which is a normal stimulus to ADH secretion) [37-39].

The incidence of selective serotonin reuptake inhibitors (SSRIs)-induced hyponatremia varies widely from 0.06% to 40%, whereas antipsychotics have a 10% incidence, antiepileptics 4.8%-41.5%, and chemotherapeutics 43% [40-46]. Drugs like angiotensin-converting enzyme inhibitors (ACEi) or angiotensin II receptor blockers (ARBs) have been found to cause hyponatremia in 48% of patients in a pilot study, more discernible in individuals >66 years of age [47]. There is also evidence of trimethoprim-sulfamethoxazole (TMP-SMX)-induced hyponatremia among the patients who were treated with >8 mg/kg/day for ≥ three days (in 72.3% of patients) [48]. Furthermore, a study that examined variations in serum chemistry among 46 patients receiving immunoglobulin (IVIG) infusions found that sodium was 4% lower than baseline [49]. A cohort of 66 patients with immune-related thrombocytopenia (ITP) who received repeated IVIG infusions presented with serum sodium levels lowered by 2.7 mmol/l in patients with normal renal function and 5.7 mmol/l in patients with acute renal dysfunction [50]. Moreover, cases of hyponatremia among patients with long-term use of amiodarone (SIADH-induced hyponatremia), propafenone, theophylline, proton pump inhibitors (omeprazole), calcium channel blockers (amlodipine), and amphetamine have been reported. Based on the underlying mechanism of idiosyncratic drug reactions causing hyponatremia, the drugs are classified into three categories [51]: drugs increasing ADH secretion from the hypothalamus, drugs increasing the sensitivity of endogenous ADH at the renal medulla, and lastly, drugs lowering the threshold for ADH secretion and resulting in reset osmostat syndrome (ROS).

An increase in ADH secretion from the hypothalamus resulting in SIADH is mostly caused by psychotropic agents like antidepressants (selective serotonin reuptake inhibitors, tricyclic antidepressants, and tetracyclic antidepressants) and antipsychotics (phenothiazines and butyrophenones). However, it must be noted that hyponatremia among emotionally disturbed or psychotic patients may be due not only to the idiosyncratic drug reactions of these medications but also to many other factors. Primary polydipsia is one of the most prominent factors in patients with psychosis, affecting nearly 7% of patients with schizophrenia [52]. Although the median onset time of total antidepressant-induced hyponatremia/SIADH was shorter than that for antipsychotic-induced hyponatremia [53], despite normal levels of ADH, some psychotropic drugs (sertraline, haloperidol), antiepileptics (carbamazepine, lamotrigine), and chemotherapeutics (cyclophosphamide) increase vasopressin V2 receptor (V2R) mRNA and aquaporin-2 (AQP2) protein expression of the inner medullary collecting duct cells, increasing water absorption even at normal or low ADH levels and leading to nephrogenic syndrome of inappropriate antidiuresis (NSIAD) [54]. Most idiosyncratic drug reactions resulting in hyponatremia involve more than one pathophysiology mentioned above. Drugs like carbamazepine or venlafaxine also lower the threshold of ADH secretion and cause dilutional hyponatremia. This is a rare form of SIADH (type C), which is difficult to correct. This condition is also known as reset osmostat syndrome [55].

In cancer patients, hyponatremia secondary to SIADH is most commonly associated with small-cell lung cancer, head and neck cancer, and other solid and hematological malignancies [56-58]. Most importantly, for individuals who have previously had remission from lymphoma treatment, a low index of suspicion for lymphoma relapse as a cause of difficult-to-treat hyponatremia is necessary [59]. In this context, chemotherapeutic agents that may lead to hyponatremia related to SIADH through different mechanisms of action include cyclophosphamide (stimulating the secretion of ADH and potentiating its action in the kidneys), vinca alkaloids (exerting a neurotoxic effect on the hypothalamic-pituitary axis, hence altering normal osmotic control of ADH secretion), cisplatin (stimulating ADH secretion, damaging renal tubules, and interfering with sodium reabsorption, resulting in salt-wasting nephropathy), as well as monoclonal antibody inhibitors of epidermal growth factor receptor (bevacizumab), tyrosine kinase inhibitors (sorafenib), and immunomodulators (pembrolizumab) [17,19,34,60,61]. It is also imperative to highlight that opioid use (morphine) in oncologic settings leads to the development of SIADH through stimulation of presynaptic µ receptors and blockage of the secretion of the inhibitor neurotransmitter gamma-aminobutyric acid (GABA). In contrast, arginine-vasopressin secretion increases [18].

The overall prevalence of hyponatremia is 2.09% among the female population and tends to increase with age [62]. In elderly patients, the prevalence of hyponatremia as an idiosyncratic drug reaction to all antidepressants is 9%, mainly in patients weighing <60kg with a previous history of hyponatremia and associated psychosis [63]. The high prevalence of multiple comorbidities and polypharmacy in older people makes them more susceptible to drug-induced hyponatremia [15]. Patients with a history of psychiatric ailments, malignancies, or elderly populations are often exposed to polypharmacy. Auriemma et al., in their study, reported elderly hospitalized patients with severe hyponatremia, with 71.4% being subject to polypharmacy [64]. The diagnosis and management are challenging in cases of idiosyncratic drug reactions causing hyponatremia, such as carbamazepine-induced hyponatremia or reset osmostat syndrome (ROS). Hyponatremia in ROS is often mild, and the patients are mostly asymptomatic. Attempting to correct the sodium increases the plasma osmolality, which increases the secretion of ADH among patients whose threshold for secretion of ADH has been lowered, resulting in a further decrease in the sodium level [51,65]. The diagnosis of ROS should only ensue after other causes of SIADH are ruled out. The water load test helps differentiate the other causes of SIADH from ROS [51]. Around 15 ml/kg of water is given orally or intravenously, and free water excretion is measured in four hours. Normal individuals and patients with ROS have been found to excrete 80% of water in four hours [51,65]. A normal fractional urea excretion is a good indicator for ROS, regardless of urine sodium or serum urea levels [65].

Endurance Exercise-Associated Hyponatremia (EAH)

Hyponatremia is very common among athletes. One of the postulated causes is excessive consumption of fluids during exercise or training during endurance sports that increase breathing and heart rate [66]. The Wilderness Medical Society Clinical Practice Guidelines and the 2015 Third International Exercise-Associated Hyponatremia Consensus Development Conference defined exercise-associated hyponatremia (EAH) as "hyponatremia occurring during or up to 24 hours after prolonged physical activity" [67,68]. A study estimated that 0.1%-1.0% of endurance athletes experience symptomatic EAH, including marathon runners, long-distance backpackers, ironman athletes, ultramarathon runners, and military service members [69]. Exercise-associated hyponatremia among athletes is primarily due to inappropriate ADH secretion, particularly the failure to suppress ADH secretion in the face of an increase in total body weight (due to increased water intake) [70]. This release of ADH leads to water retention and impaired water excretion. Frequent water intake with inappropriate water retention leads to hyponatremia. Several potential stimuli to ADH secretion among athletes include exercise-related stresses (pain), nausea, vomiting, hypoglycemia, exposure to heat, and drug use (non-steroidal anti-inflammatory drugs (NSAIDs), SSRIs). Due to muscle inflammation, IL-6 production increases after endurance exercise; a study showed a positive correlation between ADH and IL-6 production [66,70]. Sodium losses through sweat could also contribute to the development of hyponatremia, providing a hypovolemic stimulus to ADH release, which, as described above, would impair the excretion of ingested water. Exercise coupled with heat exposure for a prolonged period, particularly in non-acclimatized individuals, may contribute to EAH [71,72]. Athletes who drink copious amounts of fluid have elevated ADH levels due to increased water in their gastrointestinal (GI) tract and rapid absorption of this fluid as GI blood flow post-event increases; additionally, impaired free water excretion may lead to hyponatremia [70]. It is essential to differentiate dehydration from EAH, as isotonic or hypotonic fluids are appropriate for dehydrated athletes, but such treatment could be detrimental for an athlete with EAH. Rhabdomyolysis associated with EAH has been reported among occasional ultramarathon runners participating in races >153 km. Evidence in these reports suggests hyponatremia might increase the susceptibility to muscle cell damage [73,74].

Postoperative Hyponatremia

Loss of blood and bodily fluids, the stress response to surgery, intravenous fluid administration, blood transfusion, and the underlying surgical condition put postoperative patients at risk for electrolyte imbalances [75]. The mortality associated with postoperative hyponatremia has been estimated at 0.34%, and it is brought on by a decreased renal capacity to eliminate free water [76]. When severe, (plasma sodium concentration (PNa) <120 mmol/L), postoperative hyponatremia can result in seizures, obtundation, coma, and respiratory arrest [77-80].

Neurosurgical patients are prone to developing euvolemic hyponatremia postoperatively; in this setting, it is crucial to distinguish SIADH and cerebral salt wasting (CSW) based on the extracellular volume status of the patient. For this purpose, the N-terminal prohormone of brain natriuretic peptide (NT-pro-BNP), which is a stable biomarker with a half-life of about 120 minutes and can be stored in glass tubes for over 72 hours, is helpful as a quick and convenient assay [81,82]. With a 100% sensitivity and 66.7% specificity, NT-proBNP can accurately predict hypovolemia and a cut-off value of 125 pg/ml differentiates CSW from SIADH (with 87.50% sensitivity and 93.33% specificity) [81]. The incidence of SIADH following transsphenoidal surgeries ranges from 1.8% to 37%, and tolvaptan has been reported to yield significant sodium correction rates, notwithstanding adverse effects, therefore reducing the length of hospital stay [83-88]. Hyponatremia due to SIADH is also associated with schwannoma, owing to adrenal insufficiency and panhypopituitarism caused by an intra- or suprasellar mass [89]. Corpus callosum agenesis (CCA), a common midline defect associated with various neurological disorders and syndromes, may present with seizures, among other symptoms, thus necessitating the administration of antiepileptic medications, which have been associated with SIADH, as mentioned above [90].

Furthermore, despite SIADH being challenging to investigate due to its heterogeneity, a ratio of copeptin to urinary sodium below 30 pmol/mmol helps verify euvolemic hyponatremia; copeptin is the C-terminal fragment of the ADH hormone produced in our body, and it is thus considered a reliable surrogate of activated ADH, at the same time reflecting the intravascular volume state [91]. On this note, copeptin assessment may particularly optimize the diagnostic accuracy, management, and outcome of traumatic brain injury (TBI) in the acute phase [92]. The incidence of hyponatremia due to SIADH is also high in Cushing's disease patients as a post-surgical osmoregulatory complication, essentially central adrenal insufficiency, whereby glucocorticoid deficiency prompts the compensatory rise of arginine vasopressin (AVP) [93,94].

Other Conditions Related to Hyponatremia

**Figure 1:**
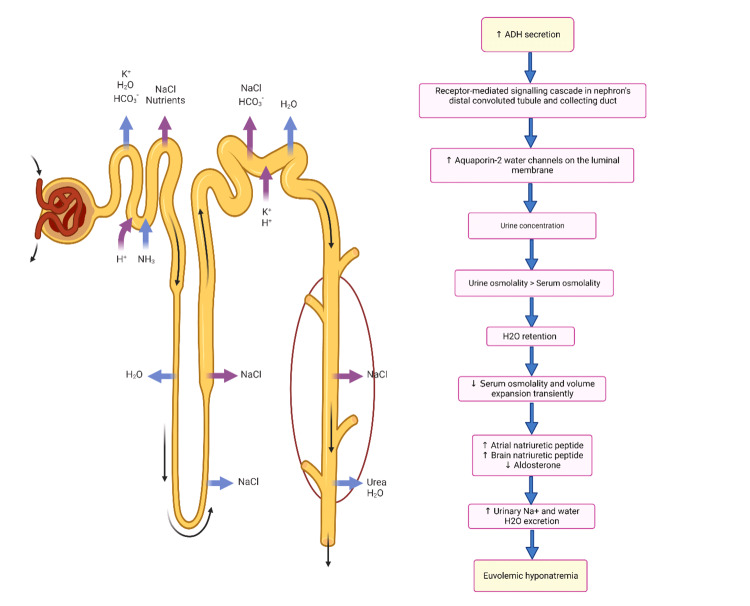
Pathophysiological mechanisms that are involved in the syndrome of inappropriate antidiuretic hormone secretion (SIADH), leading to euvolemic hyponatremia ADH: antidiuretic hormone; H_2_O: water; NaCl: sodium chloride; NH_3_: ammonia; H^+^: hydrogen; K^+^: potassium; Na^+^: sodium; HCO^3^: bicarbonate The image has been created by the authors.

In children, hyponatremia due to SIADH is linked to hypothalamic-chiasmatic tumors, meningitis, encephalitis, and sepsis [16]. Furthermore, a retrospective study found that SIADH was highly frequent in children and adolescents with brucellosis (21.9%). It was associated with elevated alanine transaminase (ALT), aspartate aminotransferase (AST), lactate dehydrogenase (LDH), C-reactive protein (CRP), blood glucose, and mean platelet volume (MPV), as well as decreased chloride, potassium, hemoglobin, albumin, and total protein, thus indicating a possible link between inflammation severity and SIADH [95]. In addition, a study by Dulger et al. prospectively evaluated 35 adult patients with brucellosis and found a SIADH frequency of 57% that was also associated with high LDH levels, high globulin levels, and low albumin [20]. Likewise, critical care patients with CNS disorders, carcinomas, and pulmonary disorders exposed to many routinely used medications are prone to hyponatremia due to SIADH [96-100]. In addition, hyponatremia caused by the human immunodeficiency virus (HIV) is also associated with SIADH [101]. Interestingly, in patients with congestive heart failure (who have a reduced blood flow resulting in nonosmotic secretion of AVP) and euvolemic hyponatremia associated with SIADH (in whom AVP secretion is not entirely suppressed even in hypoosmolality), tolvaptan is helpful in the correction of hyponatremia [102-104]. Euvolemic hyponatremia secondary to SIADH (Figure 1) has also been observed in Kawasaki disease (KD) patients as a result of nonosmotic secretion of ADH and salt wasting; hence, in these patients, infusion of hypotonic solutions with low sodium concentrations should be avoided [105].

Table 1 displays a variety of cases of hyponatremia associated with SIADH due to multiple etiologies. 

**Table 1:** Uncommon case reports and associations with SIADH ACTH: adrenocorticotropic hormone; ADH: antidiuretic hormone; CCA: corpus callosum agenesis; CNS: central nervous system; COPD: chronic obstructive pulmonary disease; COVID-19: coronavirus disease 2019; CRP: C-reactive protein; CSF: cerebrospinal fluid; CT: computed tomography; ECG: electrocardiogram; ECHO: echocardiography; EEG: electroencephalogram; GIST: gastrointestinal stromal tumor; ICIs: immune checkpoint inhibitors; IV: intravenous; LDH: lactate dehydrogenase; MRI: magnetic resonance imaging; NHL: non-Hodgkin's lymphoma; NSIAD: nephrogenic syndrome of inappropriate antidiuresis; PPI: proton pump inhibitor; PET: positron emission tomography; ROHHAD: rapid-onset obesity with hypothalamic dysfunction, hypoventilation, and autonomic dysregulation; SAC: suprasellar arachnoid cyst; SCLC: small cell lung carcinoma; SIADH: syndrome of inappropriate antidiuretic hormone; TFTs: thyroid function tests; VAT: video-assisted thoracoscopic surgery

Author, Year	Association and underlying cause of SIADH	Presentation	Diagnosis	Treatment of hyponatremia	Outcome
Meena et al. (2023) [90]	Antiepileptic medication for seizures due to CCA and hyponatremia	An eight-year-old girl with a seizure disorder and mild developmental delay under neurology follow-up	MRI, low sodium (116 mEq/L), low serum osmolarity (255 mOsm/kg), increased urinary osmolarity (890 mOsm/kg), and increased urinary sodium (75 mEq/L)	3% sodium chloride, valproate loading, fluid restriction, and tolvaptan	The patient remained seizure-free with a stable sodium level.
Schlegel (2022) [106]	Macroprolactinoma-induced SIADH	A 29-year-old woman presenting with euvolemic, hypotonic hyponatremia, normal thyroid, and glucocorticoid axes, and inappropriately concentrated urine was found to have a sellar mass with an elevated prolactin level.	Brain MRI, increased prolactin level of 193.7 ng/mL	Cabergoline and transsphenoidal resection of the mass	Rapid resolution of the mass with dopamine agonist therapy.
Mammadova et al. (2021) [107]	NSIAD with unexplained euvolemic hyponatremia	A two-month-old male presenting with hyponatremia, low plasma osmolality, relatively high urine osmolality, and low plasma renin-aldosterone levels; a one-year-old admitted with a history of oligohydramnios, four hospitalizations due to hyponatremia, and a diagnosis of epilepsy	AVPR2 gene analysis	Fludrocortisone, fluid restriction, and discontinuation of medication	Normalized serum sodium even though plasma AVP level was undetectable.
Zeybek et al. (2021) [108]	Hyponatremic newborn with SIADH and response to tolvaptan	A premature female neonate and a case of oligohydramnios was admitted to the neonatal intensive care unit (NICU) due to vomiting and neonatal convulsion on the 27^th^ day postnatally	Transfontanel ultrasonography, serum sodium (119 mmol/L), urine sodium (154 mmol/L), serum osmolality (270 mOsm/L), urine osmolality (450 mOsm/L), and plasma ADH level (21.05 pmol/L)	Drainage of the hematoma, IV hypertonic sodium infusion, fluid restriction, tolvaptan (single dose), and hypertonic saline infusion	The serum sodium level was normalized with stable neurological status.
Lindner et al. (2021) [109]	SIADH after vaccination against COVID-19	A 79-year-old male presented with weakness, fatigue, and anorexia; the patient was euvolemic despite a worsening general health condition.	Serum sodium of 117 mmol/L, serum osmolality of 241 mOsm/kg, and urea of 1.2 mmol/L with creatinine within the normal range; urine osmolality of 412 mOsm/kg, and urine sodium of 110 mmol/L	Crystalloid fluids, fluid restriction, and urea	Hyponatremia resolved following urea therapy
Chittal et al. (2021) [110]	Asymptomatic hyponatremia triggered by COVID-19 pneumonia	An 81-year-old female with a history of hypertension, on thiazide diuretic, with COVID-19 pneumonia, treated with remdesivir and dexamethasone and discharged with normal lab findings, presented to the emergency department after a fall.	Sodium of 111 mmol/L, urine sodium of 72 mmol/L, serum osmolality of 231 mOsm/kg, urine osmolality of 454 mOsm/kg	Fluid restriction, tolvaptan	Serum sodium was corrected; the patient recovered from pneumonia (severe hyponatremia later on, possibly due to the lasting effects of lung inflammation).
Patel et al. (2021) [111]	Hyponatremia triggered by ICIs	A 62-year-old Caucasian male presenting with fever, nausea, fatigue, decreased appetite, and insomnia; a medical history of metastatic melanoma; BRAFV600 mutation negative status; and currently on immunotherapy	Elevated blood urea nitrogen/creatinine ratio of 26, low urine sodium and potassium, and low serum osmolality	IV isotonic normal saline and immunotherapy with ipilimumab and nivolumab	Gradual improvement in serum sodium level
Puma et al. (2021) [112]	Tolvaptan and SAC in a child with SIADH	A two-year-old female infant was admitted to the hospital with progressively increasing head circumference and neuro-psychomotor developmental delay and was diagnosed with a large suprasellar arachnoid cyst (SAC). At three years of age, she had a seizure during non-febrile acute gastroenteritis, the acute episode, and was treated with rectal diazepam.	Brain MRI, hyponatremia (serum sodium level of 123 mEq/L), with unremarkable ABGs, electrolytes, and renal function tests	Tolvaptan, IV isotonic saline solution, 3% salts as a bolus, fluid restriction, salt, and urea supplementation	Serum sodium levels normalized years after starting therapy with tolvaptan.
Chowdhury et al. (2020) [113]	COVID-19 associated with SIADH	A 70-year-old female with a past medical history of hypertension presented with a one-day history of high-grade fever, dyspnea, and unconsciousness for four hours.	Chest X-ray, ECG, brain CT, serum sodium of 114 mEq/L, blood urea of 11.6 mg/dL, hypouricemia (2.4 mg/dL), urine osmolality of 353 mOsm/kg, low plasma osmolality of 219 mOsm/kg, urinary sodium of 34 mEq/L, random serum cortisol at 8 a.m. of 14 mcg/dL	Slow hypertonic 3% NaCl solution, nasogastric fluid restriction, supplemental oxygen, IV meropenem, thromboprophylaxis with enoxaparin, antiviral oral favipiravir, IV methylprednisolone, and oral clarithromycin	Remarkable improvement in the patient's consciousness after six hours
Saha et al. (2020) [114]	Trimethoprim-induced hyponatremia	A 72-year-old male with COPD and depression presented with progressively worsening dyspnea, requiring intubation and mechanical ventilation. Five days after therapy, the patient presented with confusion and was diagnosed with salt-losing nephropathy from trimethoprim.	Bedside ultrasound and serum sodium of 113 mmol/L	Discontinuation of trimethoprim, IV normal saline, and salt tablets	The sodium level improved within two weeks.
Rhyu et al. (2020) [115]	SIADH and lumbar CSF for a traumatic basilar skull fracture	A 31-year-old female hospitalized due to traumatic facial and skull base fractures was managed conservatively and underwent lumbar CSF drainage for six days to treat the leak.	Serum sodium of 136 mmol/L, serum osmolality of 260 mOsm/kg, urine osmolality of 482 mOsm/kg, urinary sodium of 175 mmol/L	Sodium tablets, fluid restriction, and hypertonic saline	Sodium levels normalized within 16 hours after the removal of the lumbar drain.
van der Zalm et al. (2020) [116]	PPI-induced asymptomatic hyponatremia	A 67-year-old Caucasian male presented with abdominal pain, hyponatremia, and a history of reflux esophagitis, for which he was taking omeprazole and later pantoprazole.	Serum osmolarity: 274 mOsmol/kg, urinary osmolarity: 570 mOsmol/kg, and urinary sodium: 35 mmol/L.	PPI treatment was continued, sodium levels were monitored regularly, and there was no fluid restriction.	Sodium levels remained stable over time with monitoring.
Joshi et al. (2019) [59]	Hyponatremia secondary to SIADH associated with a rare, isolated CNS relapse from a large B-cell non-Hodgkin's lymphoma (NHL).	A 76-year-old female presenting with headache and diplopia for over one month; previously, she had developed abdominal pain, a large mediastinal mass with splenic lesions, and a high LDH level.	Biopsy, PET scan, serum sodium of 116 mmol/L, low serum osmolality (232 mOsm/kg), high urine osmolality (546 mOsm/kg), and raised urine sodium of 54 mmol/L	Chemotherapy followed by radiotherapy, dexamethasone, fluid restriction, demeclocycline, and tolvaptan (single dose).	Hyponatremia responded well to tolvaptan (single dose); sodium levels normalized within two weeks.
Ioannou et al. (2018) [117]	Hyponatremia due to linezolid-induced SIADH	An 89-year-old female presenting with a complete heart block; three days before admission, she developed fatigue, generalized weakness, and a low-grade fever of 37.6°C	ECG, brain CT, serum sodium of 116 mEq/L, plasma osmolarity (243 mOsm/kg), urine osmolarity of 225 mOsm/kg, urine sodium of 93 mEq/L, serum urea (13 mg/dL), fractional excretion of urea (105.3%), fractional excretion of uric acid (25.2%), and serum uric acid of 2.8 mg/dL	Isotonic fluids and discontinuation of linezolid	The patient had remitting SIADH, even after linezolid was discontinued
Nikoomanesh et al. (2018) [118]	SIADH and small cell lung carcinoma (SCLC)	A 64-year-old Caucasian male presenting with the chief complaint of nausea and vomiting occurring frequently, with a family history of Hodgkin's lymphoma and a past medical history of smoking 20 packs per year, was diagnosed with limited-stage small cell lung cancer.	Chest X-ray, chest CT, PET scan, brain MRI Low serum osmolality (227 mOsm/kg) and high urine osmolality (579 mOsm/kg)	Fluid restriction	The patient's nausea and vomiting resolved following an improvement in sodium levels.
Song et al. (2017) [89]	SIADH associated with a mediastinal schwannoma	A 75-year-old female presenting with nausea, vomiting, and generalized weakness	Chest CT, rapid ACTH stimulation test, and biopsy with VAT	Tolvaptan	Hyponatremia completely resolved after schwannoma resection, and ADH levels decreased.
Tuli et al. (2017) [16]	Central hypothyroidism and hyponatremia	A male patient with ROHHAD syndrome (rapid-onset obesity with hypothalamic dysfunction, hypoventilation, and autonomic dysregulation)	Brain MRI and CT scan, EEG Plasma sodium of 134 mmol/L, plasma osmolality (268 mOsm/kg), urea of 33 mg/dL, creatinine (0.34 mg/dL), copeptin of 14 pmol/L, urine sodium of 96 mmol/L, and urine osmolality of 484 mOsm/kg	IV isotonic and hypertonic saline solutions with oral low-dose tolvaptan	Serum sodium levels normalized. No acute or severe symptoms were observed due to hyponatremia.
	Low-grade ganglioma and SIADH	A four-year-old female with a large sellar and suprasellar tumor with chronic euvolemic hyponatremia due to SIADH and a history of central precocious puberty, severe hypothalamic obesity, and grand mal seizure	Brain MRI and CT scan, EEG, neurosurgical biopsy, plasma sodium of 133 mmol/L, plasma osmolality of 265 mOsm/kg, urea of 30 mg/dL, creatinine of 0.30 mg/dL, copeptin of 16.6 pmol/L, urine sodium of 144 mmol/L, and a urine osmolality of 502 mOsm/kg	IV phenobarbital, hypertonic saline solution, and tolvaptan	Serum sodium levels were nearly normal after three years of treatment with tolvaptan; there was no severe hyponatremia despite the increase in the size of the tumor and the onset of central adrenal insufficiency.
	Hypothalamic-chiasmatic astrocytoma and SIADH	A five-year-old male with chronic euvolemic hyponatremia due to SIADH after neurosurgical partial removal of a hypothalamic-chiasmatic astrocytoma and a history of central precocious puberty, with symptoms of headache, nausea, asthenia, and seizures persisting after chemotherapy withdrawal	Bain MRI and CT scan, EEG, neurosurgical biopsy, serum sodium levels of 123 and 130 mmol/L	Tolvaptan and oral cortone acetate	Serum sodium levels remained stable.
Fonseca et al. (2017) [119]	SIADH with small-cell lung cancer, GIST, and colon carcinoma	A 79-year-old Caucasian female with microcytic anemia was hospitalized for hyponatremia and worsening anemia. The patient had asthenia, anorexia, and dizziness.	Chest X-ray and CT, colonoscopy, lung biopsy with fibrobronchoscopy, sodium level of 119 mmol/L, normal transaminases, CRP of 9 mg/dL, and urinary osmolality >300 mOsm/kg	Fluid restriction, furosemide, and urea with normalization of sodium	There was no resolution of SIADH, therefore, the lung tumor could still have been producing ADH.
Harsch et al. (2017) [120]	Hypertrophic pachymeningitis and SIADH	A 70-year-old female patient presented with nausea and collapsed; despite being euvolemic, pathological laboratory findings revealed hyponatremia and hypoosmolality.	Brain MRI	Moderate fluid restriction	Restoration of normal serum osmolality and sodium levels
Jung et al. (2016) [121]	Pregabalin-induced SIADH	A 69-year-old male presenting with general weakness and fever, with a severe ulcer on his right heel due to arteriosclerosis obliterans. The patient was started on pregabalin therapy for pain relief in the right heel.	Chest CT, sodium level of 138 mEq/L, reduced serum osmolality of 249 mOsm/kg, urine sodium of 91 mEq/L, and urine osmolality of 451 mOsm/kg	Ceftriaxone and clarithromycin for pneumonia, fluid restriction, saline, and discontinuation of pregabalin	Improvement in biochemical findings after stopping pregabalin therapy without any side effects
Arshad et al. (2016) [122]	SIADH induced by pharyngeal squamous cell carcinoma	A 46-year-old Caucasian female presenting with progressive dysphagia and a previous complaint of sore throat was diagnosed with streptococcal pharyngitis, for which she received antibiotics. She also had a headache associated with nausea and unintentional weight loss.	CT scan and MRI of the neck, along with a biopsy of the neck mass Sodium level of 118 meq/L, blood urea nitrogen of 4 mg/dL, and creatinine (0.56 mg/dL).	Normal saline, fluid restriction, IV furosemide, and sodium chloride tablets	Sodium levels were normalized within two weeks.
Mansoor et al. (2015) [123]	Hyponatremia and cryptococcal meningitis	A 67-year-old male with liver transplantation due to hepatitis C-related liver cirrhosis developed severe hyponatremia. The patient was treated with a normal serum sodium level, but a few days later, he presented with dizziness, confusion, ataxia, abnormal muscle movements, and leg pain.	Chest X-ray, brain MRI, lumbar puncture, hyponatremia (115 mmol/L), hyperkalemia (6.9 mmol/L), serum osmolarity of 254 mOsmol/L, urine sodium of 70 mmol/L, and urine osmolarity of 503 mOsmol/L	Amphotericin B, IV normal saline, and hypertonic saline (3%)	The patient's level of consciousness significantly improved, and his serum sodium level normalized over three weeks after starting amphotericin B therapy.
Karahan et al. (2014) [18]	Codeine-induced SIADH	A 77-year-old female presenting with a three- to four-day history of oliguria, nausea, vomiting, weakness, anorexia, dizziness, abdominal pain, constipation, and abdominal bloating. Admitted for painful lesions (shingles) on the right lumbar region.	TFTs, ECG, ECHO, and serum ADH levels were not measured. Serum sodium concentration of 112 mmol/L, serum osmolarity of 238 mOsm/L, urine sodium concentration of 55 mmol/L, and urine osmolality of 452 mOsm/kg	3% saline infusion, fluid restriction Discontinuation of codeine	Symptoms resolved
Pham et al. (2013) [124]	Hyponatremia as a rare complication of amiodarone	An 84-year-old Caucasian male with a past medical history of hypertension and diabetes was admitted due to a non-ST elevation myocardial infarction and underwent coronary artery bypass graft, developing atrial fibrillation postoperatively. The patient was started on amiodarone, and his serum sodium level decreased. The patient also presented with an altered mental status.	Serum sodium level of 105 mmol/L, TSH (0.7 µIU/mL), cortisol of 16 µg/dL, serum osmolality of 228 mOsm/kg, and urine osmolality of 251 mOsm/kg	Hypertonic (3%) saline, demeclocycline, and fluid restriction	The patient's mental status improved, and his serum sodium normalized within 10 days.

Diagnostic Approach of Hyponatremia Secondary to SIADH

A set of long-standing clinical and laboratory criteria developed by Schwartz and Bartter are still in practice for diagnosing SIAD [14]. Other causes of reduced diluting capacity, such as renal, pituitary, adrenal, thyroid, cardiac, or liver disease, must be ruled out before the diagnosis is established [14]. The standard evaluation of a suspected hyponatremic patient should be conducted through a basal metabolic panel (BMP) to confirm the level of serum sodium and other electrolytes, as well as blood glucose and blood urea nitrogen (BUN), to rule out the possibility of hyperglycemic and uremic conditions [125]. Serum osmolarity is checked in the next stage to determine the tonicity of the serum. Further evaluation of the patient depends on their clinical status. Thyroid function tests and morning cortisol levels may help exclude other causes of euvolemic hyponatremia [125]. X-ray imaging and computed tomography (CT) of the chest, help evaluate for lung diseases in cases of pneumonia or suspicion of small cell lung cancer, and CT or magnetic resonance imaging (MRI) is used in cases of subarachnoid hemorrhage, subdural hematoma, brain tumors, recent head injury, or neurologic signs, are additional tests for addressing possible underlying causes of SIADH [125].

Both low plasma osmolality and urine osmolality surpassing 100 mOsm/kg are necessary for diagnosing SIADH [103]. Euvolemic hyponatremia, serum Na+ <135 mmol/L, serum osmolality <275 mOsm/kg, urine osmolality >100 mOsm/kg, urine sodium >25 mmol/L, normal volume status, and exclusion of other causes of hyponatremia (as aforementioned) are among the diagnostic criteria for SIADH [125,126]. A trial of volume expansion is performed to differentiate between hypovolemic hyponatremia and euvolemic hyponatremia in some cases where there is ambiguity. In the case of euvolemic hyponatremia, serum sodium declines further, whereas, in hypovolemic hyponatremia, sodium correction increases [127]. Plasma uric acid and BUN are minor diagnostic cues that aid in diagnosing SIAD [128]. The detection of hypouricemia (serum uric acid < 4 mg/dL) is thus yet another indicator of the presence of SIADH, given that ADH induces urinary wasting of uric acid, resulting in low serum uric acid levels [14,127]. Around 70% of SIAD patients have uric acid < 4 mg/dl as compared to salt-depleted patients (40%) [128].

**Figure 2:**
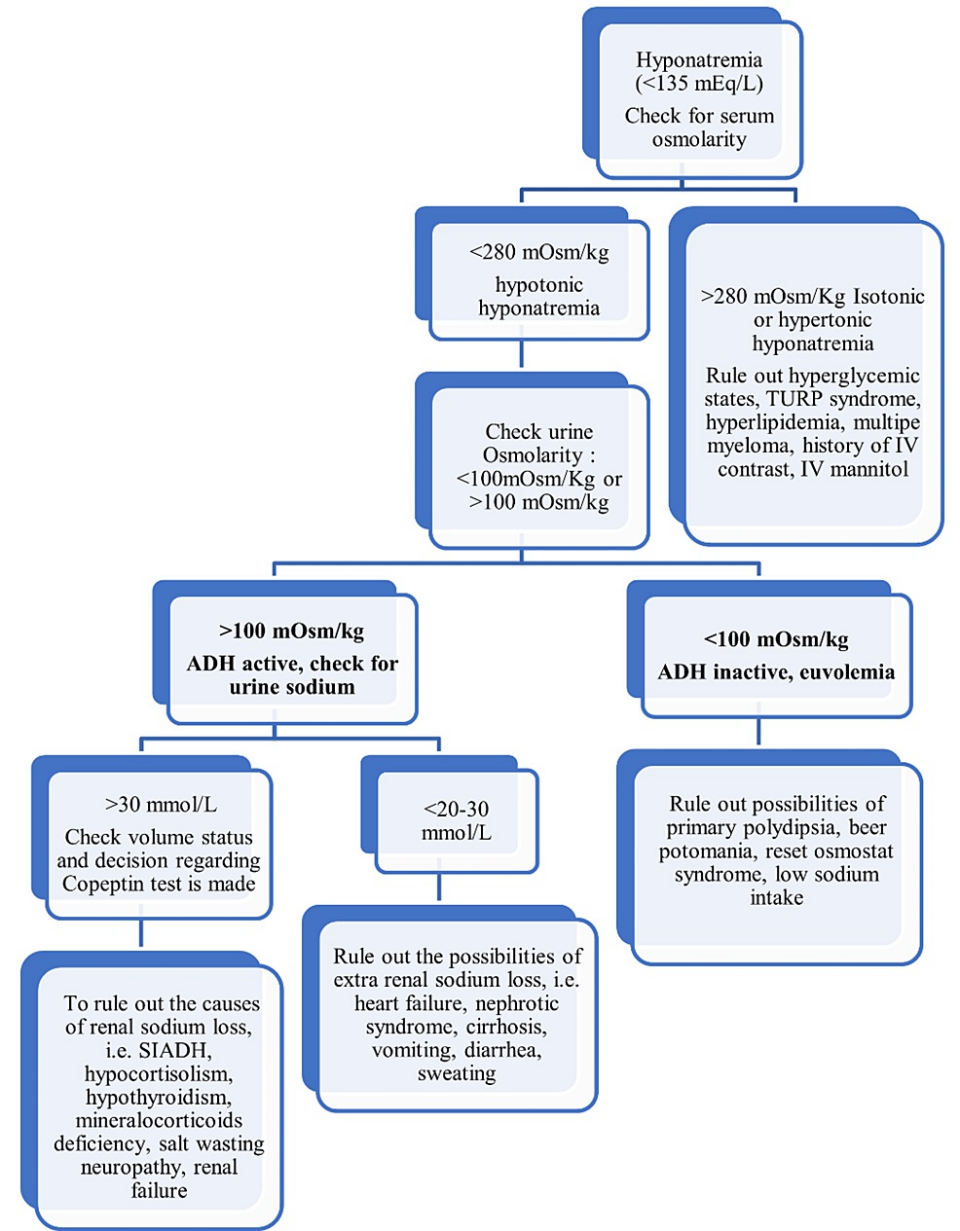
A stepwise approach to diagnosing the underlying cause of hyponatremia ADH: antidiuretic hormone; SIADH: syndrome of inappropriate antidiuretic hormone secretion; IV: intravenous; TURP: transurethral resection of the prostate The image has been created by the authors.

While urinary sodium concentration >25 mEq/L indicates renal tubular dysfunction, diuretic use, or SIADH, a concentration of <25 mEq/L denotes loss of adequate volume [95]. Therefore, differential diagnosis of euvolemic hyponatremia requires evaluation of volume status and includes postoperative excess fluid replacement and exercise-induced excess fluid intake, which must be ruled out before diagnosing patients with SIADH [70,105,129]. Additionally, ACTH deficiency and hypothyroidism should be distinguished from SIAD, as most clinical manifestations have overlapping presentations. A normal or increased ADH level is thought to be abnormal in patients with euvolemia because the body's typical response to serum hypoosmolality is to inhibit ADH secretion, an exception being nephrogenic SIADH patients with non-detectable ADH levels [77, 126,127,130]. Plasma AVP levels are undetectable due to mutations in the V2 vasopressin receptors in the nephrogenic syndrome of inappropriate antidiuresis [14]. Nephrogenic syndrome of inappropriate antidiuresis (NSIAD) and SIADH may be clinically indistinguishable; in this context, family history and lowered ADH levels may aid in the diagnosis [16]. Figure 2 illustrates a step-by-step approach to diagnosing the underlying cause of hyponatremia.

Management of Hyponatremia

**Figure 3:**
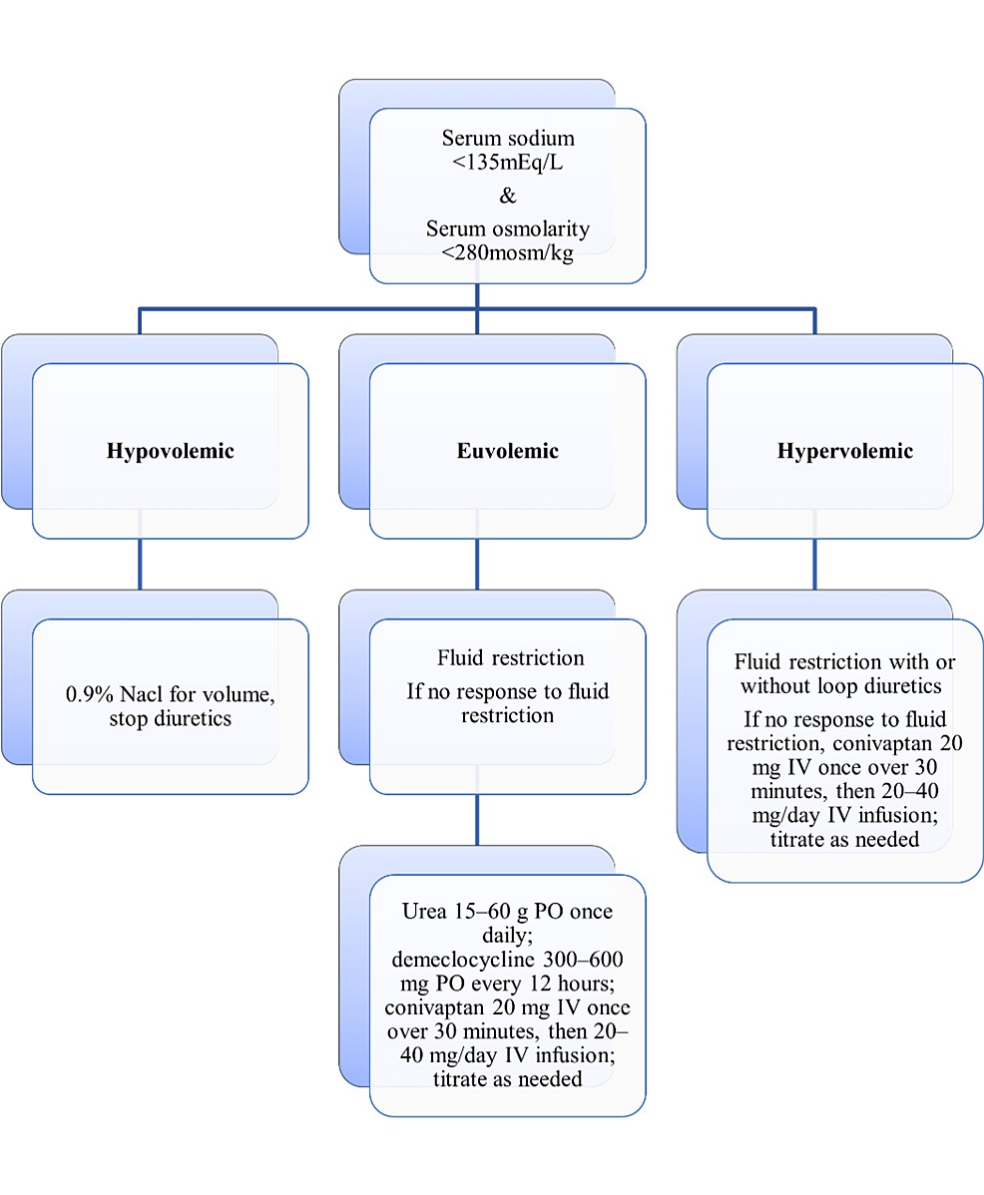
The management of hyponatremia based on volume status PO: per oral; IV: intravenous The image has been created by the authors.

The management of hyponatremia (Figure 3) depends on the underlying causes associated with volume status [8,131].

The treatment options for SIADH are numerous; therefore, clinical judgment (based on the chronicity of the hyponatremia and the neurological symptoms) and individualized risk-benefit analysis should guide therapeutic decisions [95]. Fluid restriction, saline, diuretics, and vaptans are a few treatment options associated with considerable risks and limitations [110]. Management of euvolemic hyponatremia secondary to SIADH begins with fluid restriction along with pharmacotherapy if the initial measures fail to respond [103]. The recommended fluid restriction is <1000 mL/day in patients with no signs or symptoms of SIAD or mild hyponatremia. Fluid restriction (around 500-800 cc per day) is the first-line treatment, as per many recently published articles, successfully resolving hyponatremia in approximately 60% of patients with SIADH, while adjustments are made concerning urine output and serum sodium levels following the fluid restriction, and ideal fluid intake should ideally be less than 500 mL less than daily urine output [125, 130,132-134].

Patients should be classified into acute and chronic hyponatremia to determine further steps in managing SIAD. Acute hyponatremia exceeding 48 hours is classified as chronic hyponatremia [135]. As per the new guidelines of the U.S. consensus, intravenous boluses of 3% hypertonic saline can be initiated in symptomatic hyponatremia to achieve a therapeutic goal of 4-6 mol/L over four hours compared to conventional therapy, which targets up to 12 mmol/L over 24 hours [77,136,137]. One of the risks that may come with any of the management options for hyponatremia is that of overly rapid serum Na (sNa) correction leading to osmotic demyelination syndrome (ODS) [131]. Because of the osmotic compensatory mechanisms present in the brain, in acute hyponatremia, the risk of ODS increases with very low levels of sNa (120 mEq/L) [131]. Desmopressin (1-2 mcg intravenously (IV) or subcutaneously (SC) every six to eight hours) is considered to prevent the overcorrection of sodium in patients with sodium < 120 mEq/l [77,137]. On the other hand, in chronic hyponatremia, the risk of ODS increases with the presence of hypokalemia, alcoholism, malnutrition, or advanced liver disease [103]. The minimum correction goal is 4-8 mEq/L within 24 hours, and the maximum limit (not the target) for serum sodium level increase in patients with chronic hyponatremia is 10-12 mEq/L per 24 hours [103,127]. However, with high risk for ODS, the minimum correction goal is 4-6 mEq/L within 24 hours, and the maximum correction goal is 8 mEq/L within 24 hours [103,127].

Importantly, SIADH reversal and correction of hyponatremia may be achieved by treating the underlying cause, referring to infections, cancer, nausea, or medications [125,130]. Close monitoring of the plasma sodium (PNa), initially every one to two hours, is essential for successfully managing the prevention of severe symptomatic hyponatremia and osmotic demyelination syndrome. Such monitoring will enable the timely selection of one of the following management methods or switching from one strategy to another based on treatment response. It consists of three strategies: a) proactive - which is best for individuals with PNa of 120 mEq, especially those who are at risk of overcorrection or ODS (using desmopressin acetate (DDAVP) and 3% hypertonic saline); b) reactive - if water diuresis occurs during the management of hyponatremia and the first-rate of PNa improvement is rapid enough to raise concern that it may exceed the prescribed limit of PNa correction, the reactive management method can be used that involves using DDAVP and dextrose 5% solution (D5W) to match urine output; and c) rescue - when the correction of hyponatremia has overshot the recommended safe limit, the rescue strategy should be instituted swiftly, using DDAVP and D5W which are according to the needs and the serum sodium level of the patient [103,138-140]. Urine osmolality is a crucial biomarker to determine the action of AVP. Urine osmolality >500 mOsm/kg strongly predicts a poor response to fluid restriction. The Furst formula (urine Na+ urine K/plasma sodium) strongly indicates the failure to respond to fluid restriction recommended by the U.S. and British guidelines [24,134]. If the urine/plasma electrolyte (U/P) ratio is 0.5-1.0, a fluid restriction of 1000 ml/day can be initiated. If U/P > 1.0, fluid restriction is not advisable [141]. Furthermore, urea, as a non-toxic substance, is a potential second-line treatment used in combination with low-dose diuretics and is used off-label in SIADH patients due to its cost to promote water loss by increasing the daily osmotic charge eliminated in the urine [77,142]. As such, the efficacy of urea in treating euvolemic hyponatremia secondary to SIADH is reflected in its protection against ODS [142]. Furthermore, a retrospective analysis of cancer patients with SIADH found that urea was well-tolerated and effective in correcting chronic hyponatremia within the first month of therapy in almost all (86.1%) patients [60]. The disadvantage of this drug includes poor compliance due to gastrointestinal side effects like nausea and azotemia at higher doses [103]. Off-label use of demeclocycline and low-dose loop diuretics in combination with oral salt tablets (off-label) are also treatment options for SIADH [77,103].

Vasopressin-receptor antagonists (tolvaptan, conivaptan) offer an effective therapeutic option for refractory hyponatremia secondary to SIADH when alternative treatments, such as fluid restriction, are ineffective or impractical [90]. When tolvaptan is used, the fluid restriction should be stopped to avoid overcorrection. Mechanistically, AVP drives aquaporin-2 channels to translocate to the luminal surface of cells when it binds to V2 receptors found on renal collecting ducts, eventually resulting in water retention and hyponatremia [131]. Vaptans competitively bind V2 receptors, dislodging AVP from its binding site and permitting free water excretion. Conivaptan is recommended for intravenous use in heart failure in the U.S. and Europe, whereas tolvaptan is approved for treating euvolemic hyponatremia caused by SIAD [143]. The use of vaptans for treating euvolemic hyponatremia should be considered even in pediatric patients, particularly those with severe hyponatremia symptoms and chronic conditions involved in the mechanisms leading to SIADH [16]. Hypertonic saline must be administered to rapidly correct severe acute hyponatremia with seizures or encephalopathy [90]. An observational study suggested that patients with SIADH had an increased risk for serum sodium level overcorrection with low-dose tolvaptan [144]. However, a different retrospective study revealed that low-dose (15 mg) tolvaptan is effective in correcting hyponatremia in patients with SIADH to the recommended level within current clinical guidelines, with a statistically significantly decreased tendency for overcorrection at 8 mEq/L daily [145]. In addition, conivaptan is effective in raising sNa over four days of therapy without any discernible increase in the frequency of adverse events or specific infusion-site reactions when the higher dose is used [131]. Moreover, antagonism of the vasopressin V2 receptor tends to increase electrolyte-free water excretion and sodium concentration in SIADH and edema-forming conditions such as heart failure and cirrhosis, making it an optimal therapeutic modality for many cases of hyponatremia in the critical care setting [146].

Regarding critically ill patients, tolvaptan can be used to manage hyponatremia associated with various disease states that are underlying factors in the development of SIADH, including serious infections, pneumonia, other lung diseases, and pain [96]. In individuals with SIADH and those with heart failure or cirrhosis, euvolemic hyponatremia (serum sodium 125 mEq/L) may resist correction by fluid restriction [143]. Following pituitary surgery in Cushing's disease patients, conivaptan may be an effective treatment for hyponatremia [93]. In patients with brucellosis and SIADH, hyponatremia may be resolved simply with antibiotics and fluid restriction [20]. In the Study of Ascending Levels of Tolvaptan in Hyponatremia-1 and -2 (SALT-1 and SALT-2) clinical trial, the effect of tolvaptan was studied in a randomized population diagnosed with SIAD versus placebo [147]. Results showed a remarkable increase in plasma sodium levels compared to the placebo group, with fewer adverse effects, including thirst, dry mouth, hepatotoxicity, and polyuria [148]. As a result, vasopressin receptor antagonists have emerged as a new treatment modality, as highlighted by many recently published review articles [149].

## Conclusions

Hyponatremia is a complicated electrolyte condition that presents difficult diagnostic and therapeutic hurdles. Hyponatremia involves a wide range of manifestations in the clinical setting; thus, it is imperative to distinguish different forms and underlying etiologies through volume status and plasma and urine sodium levels. Though SIAD, formerly known as SIADH, is a frequent cause of hyponatremia, additional etiologies such as hypothyroidism, adrenal insufficiency, medication responses, and other organ abnormalities should be considered. Conventional therapy with fluid restriction or hypertonic saline infusions and modern treatment modalities such as vasopressin receptor antagonists are used for patients with SIADH and other endocrine conditions. Fluid restriction is the initial treatment in acute hyponatremic patients, psychogenic polydipsia, and postoperative fluid overload cases. At the same time, hypertonic saline infusion and vasopressin receptor antagonists with close monitoring of serum sodium levels represent optimal therapeutic approaches in chronic hyponatremia patients. Although vaptans have shown positive outcomes in recent studies, their evidence is still sparse and requires more clinical trials to include them in the standardized treatment regimens for hyponatremia.
